# The Safety and Short-Term Efficacy of Aliskiren in the Treatment of Immunoglobulin A Nephropathy – A Randomized Cross-Over Study

**DOI:** 10.1371/journal.pone.0062736

**Published:** 2013-05-10

**Authors:** Cheuk-Chun Szeto, Bonnie Ching-Ha Kwan, Kai-Ming Chow, Chi-Bon Leung, Philip Kam-Tao Li

**Affiliations:** Department of Medicine and Therapeutics, Prince of Wales Hospital, The Chinese University of Hong Kong, Shatin, Hong Kong, China; Institut National de la Santé et de la Recherche Médicale, France

## Abstract

**Background:**

Laboratory research and previous study suggest that aliskiren, a direct renin inhibitor, has anti-proteinuric effects. We conducted a randomized crossover study to evaluate the anti-proteinuric effect of aliskiren in patients with immunoglobulin A (IgA) nephropathy.

**Methods:**

We studied 22 patients with biopsy-proven IgA nephropathy and persistent proteinuria despite angiotensin converting enzyme (ACE) inhibitor or angiotensin receptor blocker (ARB). Patients were randomized to either oral aliskiren 300 mg/day or placebo for 16 weeks and then crossed over to the other treatment arm after a washout period. Proteinuria, estimated glomerular filtration rate (eGFR), blood pressure, and serum potassium were monitored.

**Results:**

After aliskiren treatment, there was a significant reduction in proteinuria in 4 weeks (1.76±0.95 to 1.03±0.69 g:g-Cr, p<0.0001), which remained at a low level throughout the treatment period. There was a significant difference in proteinuria between the aliskiren and placebo groups from 4 to 16 weeks after treatment (p<0.01 for all comparisons). After aliskiren treatment, there were modest but statistically significant reductions in eGFR (57.2±29.1 to 54.8±29.3 ml/min/1.73 m^2^, p = 0.013) and diastolic blood pressure (72.6±12.3 to 66.2±11.2 mmHg, p<0.0001). None of the patient developed severe hyperkalemia (serum potassium ≥6.0 mmol/l) during the study period.

**Conclusions:**

Aliskiren has anti-proteinuric effect in patients with IgA nephropathy and persistent proteinuria despite ACE inhibitor or ARB. Further studies are needed to confirm the renal protecting effect of direct renin inhibition in chronic proteinuric kidney diseases.

**Trial Registration:**

ClinicalTrials.gov NCT00870493

## Introduction

Immunoglobulin A (IgA) nephropathy is the most common type of primary glomerulonephritis worldwide [1]. It causes end stage renal disease in 15 to 20% of individuals within 10 years of onset [2], and in 30 to 35% of individuals within 20 years of onset. Proteinuria, an elevated serum creatinine concentration, hypertension, and advanced, chronic disease in kidney biopsy predict progression [2], [3].

The optimal therapy of IgA nephropathy remains unknown. Angiotensin-converting enzyme (ACE) inhibitors or angiotensin receptor blockers (ARB) reduce proteinuria in short-term trials [4]–[7] and retard the rate of progression of renal function deterioration in chronic, proteinuric nephropathy [7], [8]. However, ACE inhibitor and ARB may not accomplish enough among high-risk patients because there exist bypass mechanisms and inhibition of the renin-angiotensin axis (RAS) is usually incomplete [9].

Renin inhibition is a new option to block the RAS at the first rate-limiting step. Preliminary data suggest a more complete suppression of the intra-renal RAAS with direct renin inhibition as compared with ARBs and ACE inhibitors [10]. Renin inhibition with aliskiren lowers blood pressure in hypertensive patients [11], [12]. There is early evidence that direct renin inhibitors may also have anti-proteinuric effect. In nondiabetic hypertensive patients, renin inhibition with remikiren leads to albuminuria reduction [13]. Recently, Persson et al [14] showed that aliskiren, the only direct renin inhibitor on the market, reduced 24-hour blood pressure, and this was associated with a reduction in albuminuria in type 2 diabetic patients. However, the efficacy of direct renin inhibitor for the treatment of non-diabetic chronic proteinuric kidney diseases has not been evaluated. The primary objective of the present study is to evaluate the safety and short-term efficacy of aliskiren, a direct renin inhibitor, on proteinuria reduction in patients with IgA nephropathy.

## Patients and Methods

The protocol for this trial and supporting CONSORT checklist are available as supporting information (see Figure S1, Checklist S1 and Protocol S1). The study was approved by our local clinical research ethics committee (Joint Chinese University of Hong Kong-New Territories East Cluster Clinical Research Ethics Committee). The study procedure was performed according to the Declaration of Helsinki. Written consent was obtained from all subjects.

### Patient Selection

This is a randomized placebo-controlled cross-over study. We recruited 22 patients with biopsy-proven IgA nephropathy from January 2010 to June 2011. Inclusion criteria were adult patients (aged 18 to 65 years) with renal biopsy-confirmed diagnosis of IgA nephropathy and require anti-hypertensive therapy, proteinuria >1 g/day (or proteinuria >1 g/g-Cr) in 3 consecutive samples despite ACE inhibitor or ARB treatment for at least 3 months, estimated glomerular filtration rate (eGFR) >30 ml/min/1.73 m^2^, and willingness to give written consent and comply with the study protocol. Renal biopsy specimens were assessed by a validated disease damage index [15] as well as the Oxford classification [16], [17]. We excluded patients who are diabetic, patients with systemic diseases that may cause IgA nephropathy or another nephropathy.

### Treatment Regimen

**Figure 1:**
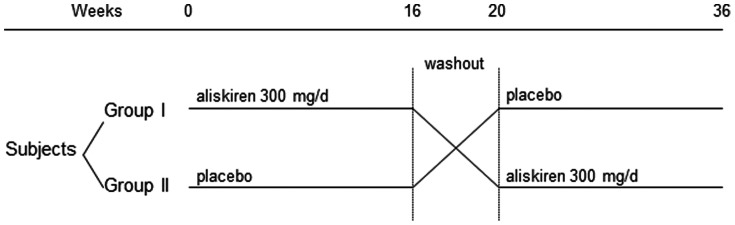
Summary of the study design and overall arrangement of treatment.

After informed consent, each patient was randomized to receive either oral aliskiren 300 mg/day or placebo for 16 weeks, followed by a washout period of 4 weeks, and then crossed over to either placebo or aliskiren for another 16 weeks (Figure 1). The appearance, packaging and labelling of the study medication and placebo were identical. Individuals were randomised by a computer-generated list, which was used for packaging of the study item and then maintained by a third party that was not involved in the conduction of the study. Marked drug packs (35 capsules for each 4 week supply) were designated for each patient. During follow up visits, study drug was dispensed by a dedicated research nurse. Both patients and investigators were blinded from the treatment allocation. Results of biochemical tests were completed before the randomisation code was broken at the end of the study.

### Follow Up Visits

Follow up visits took place at week –4 (screening), 0, 2, 4, 8, 12, 16, 20, 22, 24, 28, 32, 36 and 40 (final visit). During every visit, the following parameters were measured: body weight, blood pressure, pulse, adverse effects of treatment, complete blood picture, differential white cell count, renal function test, liver enzymes, serum calcium, phosphate, and early morning urine collection for protein-to-creatinine ratio. Renal function was determined by the estimated glomerular filtration rate (eGFR) according to a standardized formula validated in Chinese [18]. Serum fasting glucose and lipid profile were measured at 0 and 16 weeks. Prior to enrolment, all patients were taking a stable dose of ACE inhibitor or ARB. Anti-hypertensive therapy was titrated throughout the study period to maintain the blood pressure below 130/80 mmHg.

### Other Laboratory Tests

At week 0, 4, 16, 20, 24 and 36, serum level of TGF-β1 and plasma renin activity (PRA) were also measured. Patients were instructed to rest in a supine position in a quiet room for 30 minutes before each blood draw. The sample was separated within 20 minutes, and the plasma was then stored at –80°C before transport, on dry ice, to the laboratory for assay. Serum TGF-β1 levels were quantified by an enzyme-linked immunosorbent assay (ELISA) kit from the R&D Systems, Inc. (Minneapolis, MN), with a detection limit of 31.2 pg/ml. PRA was measured by a radioimmunological microassay based on angiotensin I trapping by antibody [19]. The inter- and intra-assay coefficients of the PRA assay were between 5% and 9%.

### End Points

Primary end point of the study is the change in proteinuria. Average proteinuria level at –4 and 0 week was taken as the baseline. Secondary end points include the change in eGFR and blood pressure. Information about adverse event was also recorded.

### Statistical Analysis

The sample size is estimated by the Power Analysis and Sample Size for Windows software (PASS 2000, NCSS, Kaysville, Utah). A sample size of 57 would be needed to achieve 80% power to detect a 20% reduction in proteinuria, with a significance level (alpha) of 0.05, using a two-sided paired t-test. However, because of the slow recruitment rate, only 22 patients were enrolled in the designated recruitment period, with a statistical power of 39.3% with the same assumptions.

Statistical analysis was performed by SPSS for Windows software version 15.0 (SPSS Inc, Chicago, IL). Data were expressed in mean ± SD. Data are compared by Chi-square test or Student’s t-test as appropriate between groups. Correlations between continuous variables were computed by the Pearson’s correlation coefficient. Overall effect of the treatment was determined by analysis of variance (ANOVA) for repeated measures. Post hoc analysis for individual time point was performed by paired Student’s t test with Bonferroni’s adjustment for multiple comparisons. A p value of below 0.05 was considered significant. All probabilities were two-tailed.

## Results

We recruited 22 patients. The overall study design is summarized in Figure 1. One patient withdrew consent before treatment was actually started. The results of the remaining 21 patients are analyzed. Their baseline clinical characteristics are summarized in Tables 1. There was no significant difference in the histological grading between the groups (details not shown). Amongst the 21 patients, 6 were taking lisinopril (18.3±7.5 mg/day), 4 on ramipril (11.3±6.3 mg/day), 4 on losartan (125.0±119.0 mg/day), and 7 on valsartan (108.6±50.1 mg/day). All patients were advised on dietary sodium restriction (sodium <3 g/day); 9 patients also received diuretic therapy as part of their anti-hypertensive regimen. None of the patient had been treated with corticosteroid or fish oil.

**Table 1:** Baseline characteristics of the recruited patients.

Group	Group I	Group II	P value
No. of patients	10	11	
Sex (M:F)	3∶7	4∶7	p = 0.9
Age (years)	45.0±10.9	44.0±7.7	p = 0.8
Body weight (kg)	61.8±12.0	67.9±18.5	p = 0.4
Blood pressure (mmHg)			
systolic	121.6±20.2	124.6±13.3	p = 0.7
diastolic	71.4±14.6	74.5±9.4	p = 0.6
Serum creatinine (µmol/l)	131.2±69.7	126.0±48.6	p = 0.8
eGFR (ml/min/1.73 m^2^)	59.7±33.4	55.7±24.8	p = 0.8
Urine protein (g:g-Cr)	1.89±0.84	1.94±1.01	p = 0.9

eGFR, estimated glomerular filtration rate.

### Renal Response

**Figure 2:**
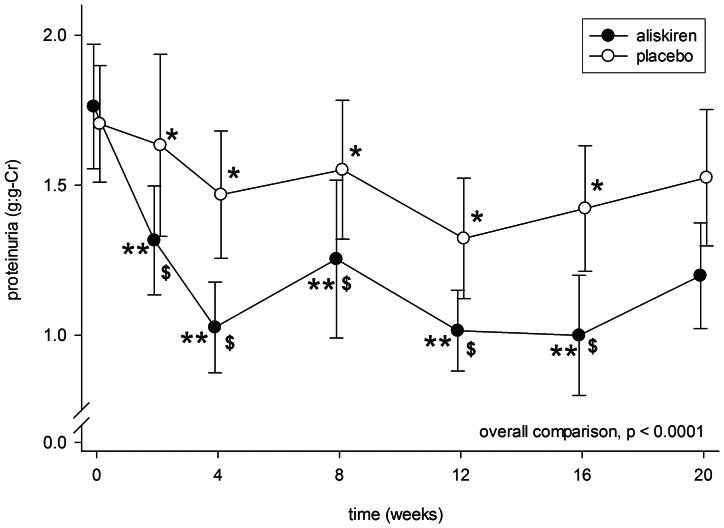
Serial change in proteinuria during the periods of aliskiren (closed circles) and placebo (open circles) treatment. Error bars denote standard error of mean. Post-hoc comparisons were performed by paired Student’s t test: *p<0.05 and **p<0.0001 as compared to baseline level; ^$^p<0.01 as compared to the period of placebo treatment. All P values were computed with Bonferroni adjustment for multiple comparisons.

After aliskiren treatment, there was a significant reduction in proteinuria in 4 weeks (1.76±0.95 to 1.03±0.69 g:g-Cr, p<0.0001), which remained at a low level throughout the treatment period (Figure 2). In contrast, proteinuria was modestly reduced after 4 weeks of placebo (1.70±0.89 to 1.47±0.97 g:g-Cr, p = 0.049) and remained static. There was a significant difference in proteinuria between the aliskiren and placebo groups from 4 to 16 weeks after treatment (p<0.01 for all comparisons).

**Figure 3:**
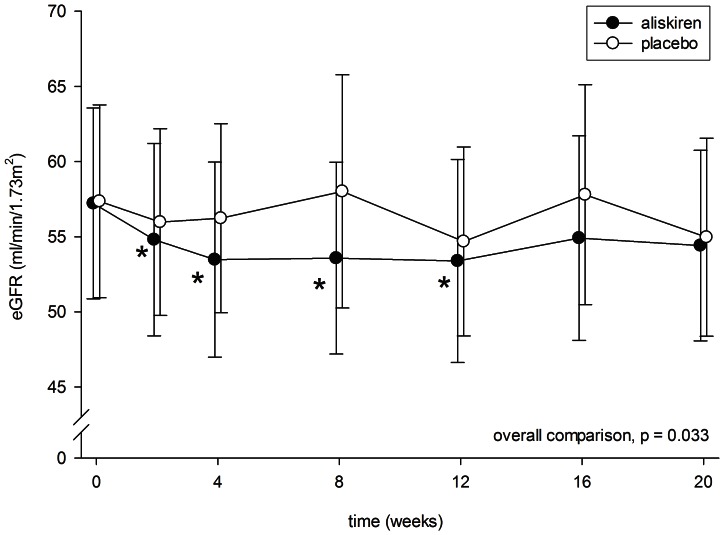
Serial change in estimated glomerular filtration rate (eGFR) during the periods of aliskiren (closed circles) and placebo (open circles) treatment. Error bars denote standard error of mean. Post-hoc comparisons were performed by paired Student’s t test: *p<0.05 as compared to baseline level. All P values were computed with Bonferroni adjustment for multiple comparisons.

After 2 weeks of aliskiren treatment, there was a modest but statistically significant decline in eGFR (57.2±29.1 to 54.8±29.3 ml/min/1.73 m^2^, p = 0.013) (Figure 3). The eGFR then remained static throughout the treatment period. In contrast, there was no significant change in eGFR throughout the period treated with placebo (see Figure 3). At 8 weeks, the aliskiren group had a slightly lower eGFR than the placebo group (53.6±29.2 vs 58.0±35.6 ml/min/1.73 m^2^, p = 0.059), but the result did not reach statistical significance. There was no significant correlation between the change in eGFR and change in proteinuria (r = 0.252, p = 0.3).

### Effect on Blood Pressure

**Figure 4:**
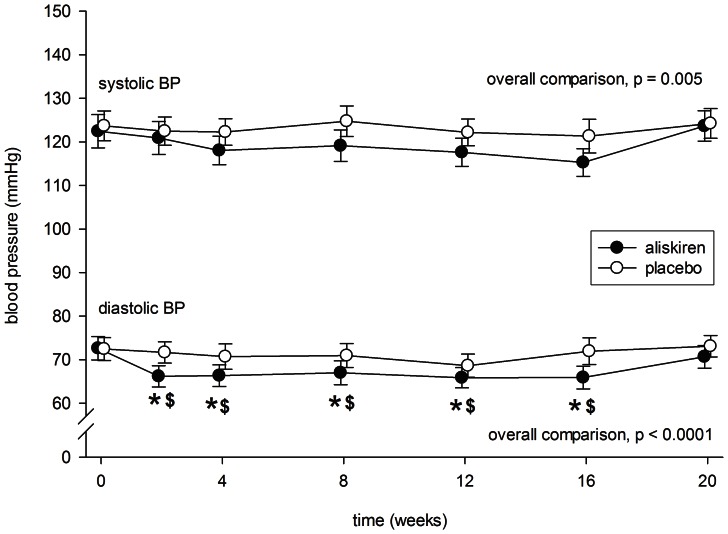
Serial change in systolic and diastolic blood pressure (BP) during the periods of aliskiren (closed circles) and placebo (open circles) treatment. Error bars denote standard error of mean. Post-hoc comparisons were performed by paired Student’s t test: *p<0.0001 as compared to baseline level; ^$^p<0.05 as compared to the period of placebo treatment. All P values were computed with Bonferroni adjustment for multiple comparisons.

After 4 weeks of aliskiren treatment, there was a modest reduction in systolic blood pressure (122.5±17.5 to 118.1±15.0 mmHg, p = 0.11) (Figure 4), but the difference did not reach statistical significance. In contrast, there was no significant change in systolic blood pressure throughout the period treated with placebo (see Figure 4). Although the aliskiren group had marginally lower systolic blood pressure than the placebo group throughout the treatment period, the difference was not statistically significant at any specific time point by post hoc analysis (details not shown).

After aliskiren treatment, there was a significant reduction in diastolic blood pressure in 2 weeks (72.6±12.3 to 66.2±11.2 mmHg, p<0.0001), which remained at that level throughout the treatment period (Figure 4). In contrast, there was no significant change in diastolic blood pressure throughout the period treated with placebo. There was a significant difference in diastolic blood pressure between the aliskiren and placebo groups from 2 to 16 weeks after treatment (p<0.05 for all comparisons).

During aliskiren treatment, there was a modest but significant correlation between the change in systolic blood pressure and change in proteinuria (r = 0.491, p = 0.024). In contrast, the change in diastolic blood pressure had no significant correlation with the change in proteinuria (r = 0.387, p = 0.083). The change in eGFR did not correlate with either systolic or diastolic blood pressure (details not shown).

### Other Biochemical Parameters

**Figure 5:**
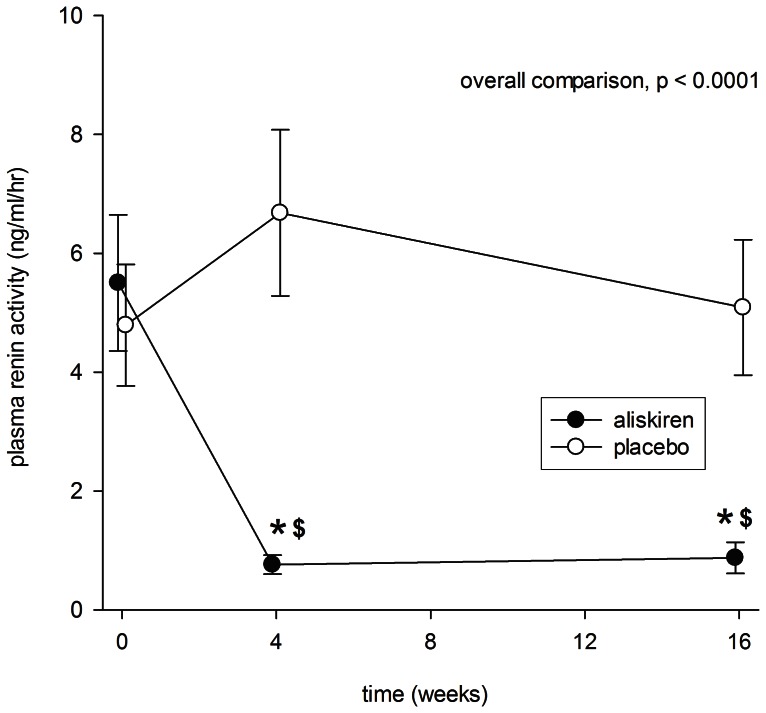
Serial change in plasma renin activity during the periods of aliskiren (closed circles) and placebo (open circles) treatment. Error bars denote standard error of mean. Post-hoc comparisons were performed by paired Student’s t test: *p<0.0001 as compared to baseline level; ^$^p<0.0001 as compared to the period of placebo treatment. All P values were computed with Bonferroni adjustment for multiple comparisons.

After 4 weeks of aliskiren treatment, there was a marked reduction in plasma renin activity (5.50±5.25 to 0.76±0.73 ng/ml/hr, p<0.0001), which remained at that level by 16 weeks (Figure 5). In contrast, plasma renin activity remained static throughout the period treated with placebo. Plasma renin activity was significantly lower in the aliskiren group than the placebo group at 4 and 16 weeks of treatment (p<0.001 for both comparisons). The reduction in plasma renin activity during aliskiren treatment did not correlate with the change in proteinuria, eGFR, systolic or diastolic blood pressure (details not shown).

During the study period, there was no significant change in serum sodium level, liver enzymes, fasting glucose or lipid profile (details not shown). There was also no significant change in serum TGF-β level during the study period (details not shown).

### Adverse Effects

**Figure 6:**
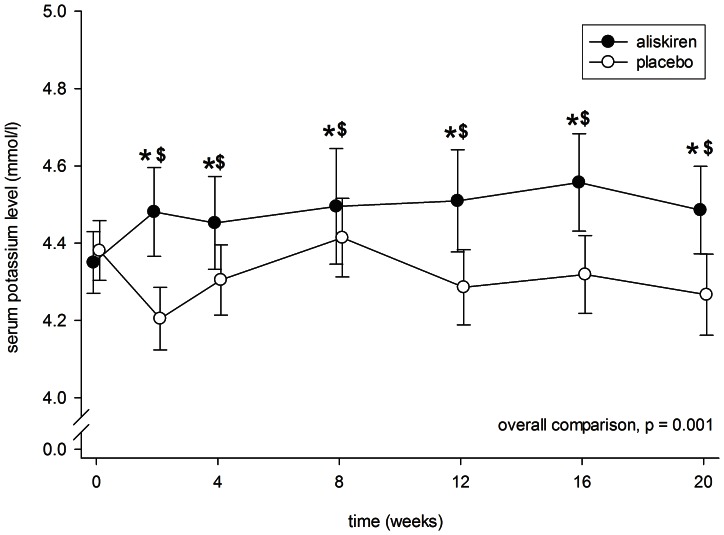
Serial change in serum potassium level during the periods of aliskiren (closed circles) and placebo (open circles) treatment. Error bars denote standard error of mean. Post-hoc comparisons were performed by paired Student’s t test: *p<0.001 as compared to baseline level; ^$^p<0.001 as compared to the period of placebo treatment. All P values were computed with Bonferroni adjustment for multiple comparisons.

The period of aliskiren treatment had a higher serum potassium level than the placebo period at 2, 12 and 16 weeks (p<0.01 for all comparisons). (Figure 6) During the period of aliskiren treatment, 4 patients had a total of 8 episodes of hyperkalemia; during the period of placebo, 1 patient had one episode of hyperkalemia. All responded to oral sodium bicarbonate and advise on low potassium diet. None of the patient developed severe hyperkalemia (serum potassium ≥6.0 mmol/l) during the study period.

In general, aliskiren was well tolerated. Three patients reported dizziness, 2 during aliskiren treatment and 1 during the placebo phase. Another patient from reported headache during aliskiren treatment. None of the patients had severe adverse effect that required hospital admission or discontinuation of the study medicine.

## Discussion

In the present study, we found that in patients with IgA nephropathy and persistent proteinuria despite ACE inhibitor or ARB therapy, aliskiren can effectively reduce proteinuria. Our study indicates that, in additon to diabetic nephropathy, direct renin inhibitor also has anti-proteinuric effect on non-diabetic glomerular disease. In general, aliskiren is very well tolerated, and its effects on blood pressure, renal function, and serum potassium level are relatively modest.

The anti-proteinuric effect of aliskiren we observed is similar to previous reports. For example, in an uncontrolled study on IgA nephropathy, Tang et al [20] reported that after 14 weeks of aliskiren treatment, proteinuria decreased from 1.73 to 1.27 g:g-Cr. In patients with type 2 diabetes and nephropathy on losartan, treatment with aliskiren for 12 months reduced the mean urinary protein-to-creatinine ratio by 26.3% [21]. More recently, Chen et al [22] also reported that add-on treatment of aliskiren reduced proteinuria and attenuated the decline in renal function in non-diabetic CKD patients who were receiving ARB. Although the magnitude of proteinuria reduction seems consistent in all these, it is interesting to note that proteinuria appears to be reduced within 4 weeks of treatment in the reports of Persson et al [14] as well as our present study, while it takes 8 weeks in the report of Chen et al [22], and 14 weeks in the study by Tang et al [20]. The reason of this discrepancy is unknown.

In the present study, we observed that proteinuria also reduces significantly during the period of placebo treatment, although the degree of reduction was less as compared to the period during aliskiren treatment. Our findings are in line with previous reports. For example, Parving et al noted that in type 2 diabetic patients with nephropathy, proteinuria was reduced by 50% or more in 12.5% of patients treated with placebo [21]. Previous studies on diabetic nephropathy also showed that intensive care received by patients in a clinical trial setting also reduced the rate of decline in renal function, presumably by the optimization of all risk factors [23].

We observe a modest but statistically significant reduction in blood pressure during the period of aliskiren treatment. The magnitude of blood pressure reduction is also consistent with previous reports [20], [21]. In theory, the anti-proteinuric effect of aliskiren could be the result of its blood pressure lowering effect. However, we found no correlation between the degree of blood pressure reduction and the decrease in proteinuria. Previous studies on diabetic nephropathy showed that the antiproteinuric effects of aliskiren were independent of baseline blood pressure [24]. Since this is a crossover study, it could be argued that the anti-proteinuric effect during the phase of placebo treatment could represent a carry-over effect of recent aliskiren treatment. However, the anti-proteinuric effect during the phase of placebo treatment was actually observed irrespective to the sequence of treatment allocation (details not shown). Based on the result of a previous study [14], a washout period of 4-weeks would be sufficient and baseline proteinuria level could be re-established.

Contrary to two previous reports [20], [25], we did not observe any reduction in serum TGF-β level in response to aliskiren treatment. It is interesting to note that our patients had much lower pre-treatment serum TGF-β levels (around 16 pg/ml) than those in Tang’s study (25 to 30 pg/ml) [20], suggesting some difference in patient selection between the studies.

In the present study, hyperkalemia was uncommon and mild during aliskiren treatment. It should, however, be noted that most of our patients had stage 2 to 3 CKD, and the risk of hyperkalemia should be considerably higher if the baseline renal function is worse. Previous studies on diabetic nephropathy reported that hyperkalemia developed in 22.5% of patients treated with aliskiren [26]. A recent meta-analysis showed that aliskiren plus ACE inhibitors or ARB increases risk for hyperkalemia more than monotherapy with any of these agents [27].

We find that short term aliskiren treatment is well tolerated. Currently the efficacy and safety of aliskiren is best tested in two clinical trials in heart failure, namely, the Aliskiren Trial of Minimizing OutcomeS for Patients with HEart failure (ATMOSPHERE) [28] and the Aliskiren Trial on Acute Heart Failure Outcomes (ASTRONAUT) [29]. However, on 20 December 2011, the Aliskiren Trial In Type 2 Diabetes Using Cardio-Renal Disease Endpoints (ALTITUDE) [30], was stopped on the recommendation after the second interim efficacy analysis [31], [32]. In this study, aliskiren or placebo was randomly assigned as an adjunct to an ACE inhibitor or ARB to type 2 diabetic patients who are at high risk for cardiovascular and renal events. Although the primary and secondary renal end points were similar between the groups after a median follow-up of 32.9 months, and the reduction in urinary albumin-to-creatinine ratio was greater in the aliskiren group, the proportions of patients with hyperkalemia and hypotension were both significantly higher in the aliskiren than the placebo group [32]. A similar problem has also been reported by the ONTARGET study, which used dual blockade of the renin-angiotensin system by combination therapy of ACE inhibitor and ARB [33]. The long term safety and efficacy of vigorous inhibition of the renin-angiotensin axis deserves further studies.

There are a number of limitations of our study. The sample size of the present study was small and we recruited patients, on average, over 5 years after their renal biopsy. There was, as a result, a possible selection bias that we enrolled patients with persistent proteinuria but relatively stable function, while the subgroup of IgA nephropathy patients with rapidly progressive disease was not represented in our study population.

More importantly, the duration of study was short and the long term efficacy of aliskiren in reducing proteinuria needs to be confirmed. We observed a “rebound” in proteinuria soon after aliskiren treatment was discontinued (see Figure 2). Because of the short follow up and crossover design of our study, we cannot make any conclusion on the effect of aliskiren on the rate of renal function decline.

In summary, our study showed that aliskiren has anti-proteinuric effect in patients with IgA nephropathy and persistent proteinuria despite ACE inhibitor or ARB therapy. Further studies are needed to confirm the renal protecting effect of direct renin inhibition in chronic proteinuric kidney diseases.

## Supporting Information

Figure S1 **CONSORT Flow Diagram.**
(TIF)

Protocol S1 **Trial Protocol.**
(DOC)

Checklist S1 **CONSORT 2010 Checklist of Information for a Randomized Trial.**
(DOC)
