# Supplementary figures and images for: The Safety and Short-Term Efficacy of Aliskiren in the Treatment of Immunoglobulin A Nephropathy – A Randomized Cross-Over Study
Source: PLoS One. 2013 May 10;8(5):e62736. doi: 10.1371/journal.pone.0062736 (PMC3651209; doi:10.1371/journal.pone.0062736)

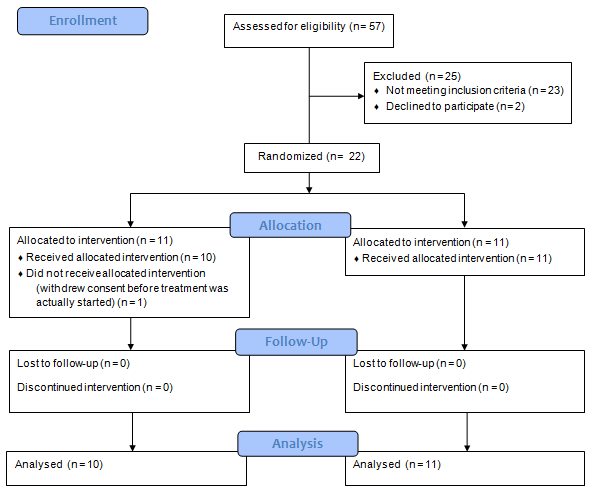

Supplement: Figure S1 — CONSORT Flow Diagram. (TIF) [file pone.0062736.s001.tif]
