# Supplementary material for: The Safety and Short-Term Efficacy of Aliskiren in the Treatment of Immunoglobulin A Nephropathy – A Randomized Cross-Over Study
Source: PLoS One. 2013 May 10;8(5):e62736. doi: 10.1371/journal.pone.0062736 (PMC3651209; doi:10.1371/journal.pone.0062736)
Supplement: Protocol S1 — Trial Protocol. (DOC) [file pone.0062736.s002.doc]

The Safety and Short-Term Efficacy of Aliskiren in the Treatment of Immunoglobulin A Nephropathy – A Randomized Cross-Over Study

Principal Investigator:

LI, Kam Tao Philip, Consultant & Honorary Professor

Co-Investigator:

SZETO Cheuk Chun, Professor

Department of Medicine & Therapeutics, The Chinese University of Hong Kong, Prince of Wales Hospital, Shatin, Hong Kong.

Protocol: AIgA / CRE-2009.057-T

Protocol date: 23 February 2009

ABSTRACT

Immunoglobulin A (IgA) nephropathy is the most common type of primary glomerulonephritis in the world. Current treatment with angiotensin converting enzyme (ACE) inhibitor and angiotensin receptor blocker (ARB) is not entirely effecive. Aliskiren, a direct renin inhibitor, acts on the rate limiting step of the renin-angiotensin axis. In addition to lowering the blood pressure, recent study in diabetic nephropathy suggests an independent anti-proteinuric effect. We plan to conduct a a randomized placebo-control cross-over study to evaluate the safety and efficacy of aliskiren in the treatment of IgA nephropathy. We plan to recruit 57 patients with biopsy-proven IgA nephropathy and persistent proteinuria despite conventional therapy. They will be randomized to aliskiren for 16 weeks or no treatment, followed by cross over to the other arm after a washout period. Proteinuria, albuminuria, renal function, serum and urinary markers will be quantified. This study will explore the potential anti-proteinuric effect of aliskiren in the treatment of IgA nephropathy, which has no specific treatment at present.

BACKGROUND

Immunoglobulin A (IgA) nephropathy is the most common type of primary glomerulonephritis worldwide [1]. It causes end stage renal disease in 15 to 20% of individuals within 10 years of onset [2], and in 30 to 35% of individuals within 20 years of onset. Proteinuria, an elevated serum creatinine concentration, hypertension, and advanced, chronic disease in kidney biopsy predict progression [2, 3].

The optimal therapy of IgA nephropathy remains unknown. Angiotensin-converting enzyme (ACE) inhibitors or angiotensin receptor blockers (ARB) reduce proteinuria in short-term trials [4-7] and retard the rate of progression of renal function deterioration in chronic, proteinuric nephropathy [7,8]. However, ACE inhibitor and ARB may not accomplish enough among high-risk patients because there exist bypass mechanisms and inhibition of the renin-angiotensin axis (RAS) is usually incomplete [9].

Renin inhibition is a new option to block the RAS at the first rate-limiting step. Preliminary data suggest a more complete suppression of the intra-renal RAAS with direct renin inhibition as compared with ARBs and ACE inhibitors [10]. Renin inhibition with aliskiren lowers blood pressure in hypertensive patients [11,12]. There is early evidence that direct renin inhibitors may also have anti-proteinuric effect. In nondiabetic hypertensive patients, renin inhibition with remikiren leads to albuminuria reduction [13]. Recently, Persson et al [14] showed that aliskiren, the only direct renin inhibitor on the market, reduced 24-hour blood pressure, and this was associated with a reduction in albuminuria in type 2 diabetic patients. However, the efficacy of direct renin inhibitor for the treatment of non-diabetic chronic proteinuric kidney diseases has not been evaluated.

Although blockers of the RAS reduce blood pressure, their therapeutic benefit in proteinuric kidney diseases is generally believed to be the result of RAS inhibition. Along this line, human study of intra-renal ACE and type 2 ACE (ACE2) expression has long been hampered by the need of renal biopsy. In the last few years, with the development of reliable RNA extraction technique from urinary sediment and reverse transcription real-time quantitative polymerase chain reaction (RT-QPCR), measurement of mRNA expression in urinary sediment has become an emerging tool for the study of kidney diseases [15,16]. We recently showed that in the patients with diabetic nephropathy treated with ACE inhibitor, there was a significant inverse correlation between the degree of proteinuria and rate of renal function decline and urinary ACE2 expression [17], and urinary expression of synaptopodin was lower after 12 weeks of ACE inhibitor and ARB combination therapy [18], suggesting a reduction in urinary podocyte loss. Taken together, gene expression study of urinary sediment may provide insight to the status of intra-renal renin-angiotensin system.

OBJECTIVE

The primary objective of the present study is to evaluate the safety and short-term efficacy of aliskiren, a direct renin inhibitor, on proteinuria reduction in patients with IgA nephropathy. The secondary objective is to evaluate the effect of aliskiren treatment on the serum cytokine and urinary gene expression profile in patients with IgA nephropathy.

PATIENTS AND METHODS

This is an placebo-controlled cross-over study. We plan to recruit 57 patients with biopsy-proven IgA nephropathy.

# Inclusion criteria

- aged 18-65 years
- requires anti-hypertensive therapy
- renal biopsy confirmed the diagnosis of IgA nephropathy
- proteinuria > 1 g/day (or proteinuria > 1 g/g-Cr) in 3 consecutive samples within 12 weeks despite ACE inhibitor or ARB treatment for at least 3 months
- estimated glomerular filtration rate > 30 ml/min/1.73m^2^
- willingness to give written consent and comply with the study protocol

# Exclusion criteria

- Patients who are diabetic, and patients with systemic diseases that may cause IgA nephropathy or another nephroapthy.
- Pregnancy, lactating or childbearing potential without effective method of birth control
- Severe gastrointestinal disorders that interfere with their ability to receive or absorb oral medication
- History of malignancy, including leukemia and lymphoma within the past 2 years
- Systemic infection requiring therapy at study entry
- Any other severe coexisting disease such as, but not limited to, chronic liver disease, myocardial infarction, cerebrovascular accident, malignant hypertension
- History of drug or alcohol abuse within past 2 years
- Participation in any previous trial on aliskiren or other renin inhibitor
- Previous treatment with fish oil, steroid, cytotoxic agents, or aldosterone antagonist
- History of treatment with other drugs that may affect proteinuria within past 2 years
- Patients receiving treatment of corticosteroid
- On other investigational drugs within last 30 days
- History of a psychological illness or condition such as to interfere with the patient’s ability to understand the requirement of the study
- History of non-compliance
- Known history of sensitivity or allergy to aliskiren or other renin inhibitor

# Treatment regimen and dosage adjustment

After informed consent, each subject will be randomized to receive either oral aliskiren 300 mg/day or placebo for 16 weeks, followed by a washout period of 4 weeks, then crossed over to either placebo or aliskiren for another 16 weeks:


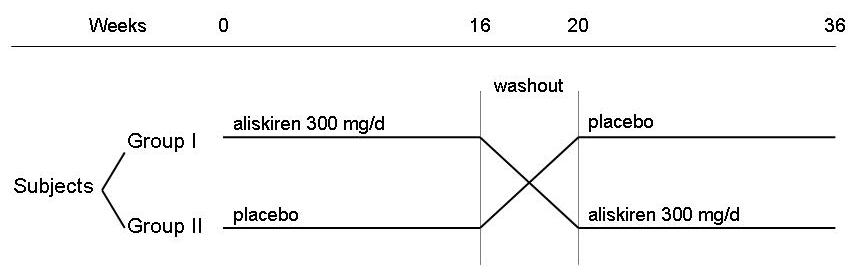


The appearance, packaging and labelling of the study medication and placebo would be identical. Individuals were randomised by a computer-generated list, which is used for packaging of the study item and then maintained by a third party that is not involved in the conduction of the study. Marked drug packs (35 capsules for each 4 week supply) will be designated for each patient. Both patients and investigators will be blinded. Results of biochemical tests will completed before the randomisation code is broken at the end of the study.

Based on the result of published study [Persson F, Rossing P, Schjoedt KJ, Juhl T, Tarnow L, Stehouwer CD, Schalkwijk C, Boomsma F, Frandsen E, Parving HH. Time course of the antiproteinuric and antihypertensive effects of direct renin inhibition in type 2 diabetes. Kidney Int 2008; 73: 1419-1425], a washout period of 4-weeks would be sufficient and baseline proteinuria level could be re-established.

During follow up visits, aliskiren will be dispensed by a dedicated research nurse; clinicians will be blinded from the treatment allocation.

*Concomitant therapy*

Prior to enrollment, all of the patients will be stable while receiving ACE inhibitor or ARB. Anti-hypertensive therapy will be titrated throughout the study period to maintain the blood pressure below 130 / 80 mmHg.

*Visit schedule*

Follow up visits will take place according to the following schedule:

- week –4 (screening)
- weeks 0, 2, 4, 8, 12, 16, 20, 22, 24, 28, 32, 36 and 40 (final visit)

During every visit, the following parameters will be measured: body weight, blood pressure, pulse, adverse effects of treatment, complete blood picture, differential white cell count, renal function test, liver enzymes, serum calcium, phosphate, and early morning urine collection for protein-to-creatinine and albumin-to-creatinine ratio. Renal function is determined by the estimated glomerular filtration rate (GFR) according to a standardized formula validated in Chinese [19]. Serum fasting glucose and lipid profile will be measured at 0 and 16 weeks. Proteinuria will be measured by a turbidimetric technique with the Modular Analytics (Roche Diagnostics, Switzerland). Urinary creatinine (Jaffe's kinetic method) and albumin (immunoturbidimetry method) were also measured on the Hitachi 911 analyzer (Boerhringer Mannheim, Mannheim, Germany), using reagent kits supplied by the manufacturer.

During week 0, 4, 16, 20, 24 and 36, whole stream early morning urine will be collected for the study of gene expression in urinary sediment. The method of specimen handling has been described in our previous studies [16-18]. Based on these studies, the target genes to be quantified include nephrin, podocin, synaptopodin, ACE, ACE2, transforming growth factor beta-1 (TGF-β1), connective tissue growth factor (CTGF), and hepatocyte growth factor (HGF). Urine supernatant will also be saved for the assay of TGF-β1, CTGF and HGF by standard ELISA. At the same time points, we will also serum level of C-reactive protein (CRP), TGF-β1, and angiotensin II will be measured by ELISA, as well as plasma renin activity (PRA) by conventional methods.

*End points*

Primary end point of the study is the change in the degree of proteinuria and albuminuria. Average levels proteinuria and albuminuria levels at –4 and 0 week will be taken as the baseline. Secondary end points include the rate of decline of estimated GFR (as determined by the least square method), and change in serum and urinary inflammatory markers.

# Adverse events

Information about every adverse event will be collected and recorded. An adverse event is any undesirable symptom or medical condition occurring after starting the study medication, whether considered drug-related or not.

## Patient withdrawal

The patient will be withdrawn from the study for:

- death
- doubling of baseline serum creatinine level
- pregnancy
- any other intolerable adverse events
- significant non-compliance with the protocol
- the desire of the patient to withdraw from the study

All female patients will be advised on taking effective contraceptive measures during the study period.

## Statistical analysis

Data will be expressed in mean ± SD. The change in proteinuria, renal function and other inflammatory markers before and after treatment will be compared by paired Student’s t test or Wilcoxon’s matched-pairs signed-rank test as appropriate. Calculation will be performed by SPSS for Windows software version 11.5 (SPSS Inc, Chicago, IL).

JUSTIFICATION

The sample size is estimated by the Power Analysis and Sample Size for Windows software (PASS 2000, NCSS, Kaysville, Utah). We expect baseline proteinuria to be around 2.0 ± 0.75 g:g-Cr [20]. Given that in the recently published AVOID study [21] aliskiren led to a 20% additional proteinuria reduction on top of losartan 100 mg, a sample size of 57 would be needed to achieve 80% power, with a significance level (alpha) of 0.05, using a two-sided paired t-test.

TIMETABLE OF WORK

It takes 24 months to complete the study:

Enrollment of case September 2009 to November 2010

Follow up of case December 2010 to August 2011

Analysis and write up report September 2011 to December 2011

REFERENCE

1. D’Amico G. The commonest glomerulonephritis in the world: IgA nephropathy. Q J Med 1987; 245: 709-727.
2. Li PKT, Ho KKL, Szeto CC, Yu LM, Lai FM. Prognostic Indicators of IgA Nephropathy in the Chinese – Clinical and Pathological Perspectives. Nephrol Dial Transplant 2002; 17:64-69
3. Haas M. Histologic subclassification of IgA nephropathy: a clinico-pathologic study of 244 cases. Am J Kidney Dis 1997; 829-842.
4. Maschio G, Cagnoli L, Claroni F, Fusaroli M, Rugiu C, Sanna G, Sasdelli M, Zuccala A, Zucchelli P. ACE inhibition reduce proteinuria in normotensive patients with IgA nephropathy: a multicentre, randomized, placebo-controlled study. Nephrol Dial Transplant 1994; 9: 265-269.
5. Russo D, Pisani A, Balletta MM, De Nicola L, Savino FA, Andreucci M, Minutolo R. Additive anti-proteinuric effect of converting enzyme inhibitor and losartan in normotensive patients with IgA nephropathy. Am J Kidney Dis 1999; 33: 851-856.
6. Perico N, Remuzzi A, Sangalli F, Azzollini N, Mister M, Ruggenenti P, Remuzzi G. The antiproteinuria effect of angiotensin antagonsim in human IgA nephropathy is potentiated by indomethacin. J Am Soc Nephrol 1998; 9: 2308-2317.
7. Li PK, Leung CB, Chow KM, Cheng YL, Fung SK, Mak SK, Tang AW, Wong TY, Yung CY, Yung JC, Yu AW, Szeto CC. Hong Kong study using valsartan in IgA nephropathy (HKVIN): a double-blind, randomized, placebo-controlled study. Am J Kidney Dis 2006; 47: 751-760.
8. Ruggenenti P, Perna A, Gherardi G, Benini R, Remuzzi G. Chronic proteinuric nephropathies: outcomes and response to treatment in a prospective cohort of 352 patients with different patterns of renal injury. Am J Kidney Dis 2000; 35: 1155-1165.
9. Staessen JA, Li Y, Richart T. Oral renin inhibitors. Lancet. 2006; 368: 1449-1456.
10. Fisher NDL, Hollenberg NK. Renin inhibition: what are the therapeutic opportunities? J Am Soc Nephrol 2005; 16: 592-599
11. Stanton A, Jensen C, Nussberger J et al. Blood pressure lowering in essential hypertension with an oral renin inhibitor, aliskiren. Hypertension 2003; 42: 1137-1143
12. Gradman AH, Schmieder RE, Lins RL et al. Aliskiren, a novel orally effective renin inhibitor, provides dose-dependent antihypertensive efficacy and placebo-like tolerability in hypertensive patients. Circulation 2005; 111: 1012-1018
13. van Paassen P, de Zeeuw D, Navis G et al. Renal and systemic effects of continued treatment with renin inhibitor remikiren in hypertensive patients with normal and impaired renal function. Nephrol Dial Transplant 2000; 15: 637-643.
14. Persson F, Rossing P, Schjoedt KJ, Juhl T, Tarnow L, Stehouwer CD, Schalkwijk C, Boomsma F, Frandsen E, Parving HH. Time course of the antiproteinuric and antihypertensive effects of direct renin inhibition in type 2 diabetes. Kidney Int 2008 Mar 12; [Epub ahead of print]
15. Wang G, Szeto CC. Quantification of gene expression in urinary sediment for the study of renal diseases. Nephrology (Carlton) 2007; 12: 494-499.
16. Szeto CC, Chow KM, Lai KB, Szeto CY, Chan RW, Kwan BC, Chung KY, Li PK, Lai FM. mRNA expression of target genes in the urinary sediment as a noninvasive prognostic indicator of CKD. Am J Kidney Dis 2006; 47: 578-586.
17. Wang G, Lai FM, Lai KB, Chow KM, Kwan CH, Li KT, Szeto CC. Urinary mRNA expression of ACE and ACE2 in human type 2 diabetic nephropathy. Diabetologia 2008 Apr 4 [Epub ahead of print].
18. Wang G, Lai FM, Lai KB, Chow KM, Kwan BC, Li PK, Szeto CC. Urinary messenger RNA expression of podocyte associated molecules in patients with diabetic nephropathy treated by angiotensin converting enzyme inhibitor and angiotensin receptor blocker. Eur J Endocrinol 2008; 158: 317-322.
19. Ma YC, Zuo L, Chen JH, Luo Q, Yu XQ, Li Y, Xu JS, Huang SM, Wang LN, Huang W, Wang M, Xu GB, Wang HY. Modified glomerular filtration rate estimating equation for Chinese patients with chronic kidney disease. J Am Soc Nephro. 2006; 17: 2937-2944.
20. Szeto CC, Chow KM, Kwan BC, Chung KY, Leung CB, Li PK. Oral calcitriol for the treatment of persistent proteinuria in immunoglobulin A nephropathy: an uncontrolled trial. Am J Kidney Dis 2008; 51:724-31
21. Parving HH, Persson F, Lewis JB, Lewis EJ, Hollenberg NK. Aliskiren combined with losartan in type 2 diabetes and nephropathy. N Engl J Med 2008; 358: 2433-2446

SUMMARY OF WORK

| Visit | Screen | 1 | 2 | 3 | 4 | 5 | 6 | 7 | 8 | 9 | 10 | 11 | 12 | 13 |
| --- | --- | --- | --- | --- | --- | --- | --- | --- | --- | --- | --- | --- | --- | --- |
| Week | -4 | 0 | 2 | 4 | 8 | 12 | 16 | 20 | 22 | 24 | 28 | 32 | 36 | 40 |
| Inclusion/exclusion criteria | ✓ | ✓ |  |  |  |  |  |  |  |  |  |  |  |  |
| Written informed consent |  | ✓ |  |  |  |  |  |  |  |  |  |  |  |  |
| Pregnancy test* |  | ✓ |  |  |  |  |  |  |  |  |  |  |  |  |
| Drug dispensing |  | ✓ | ✓ | ✓ | ✓ | ✓ |  | ✓ | ✓ | ✓ | ✓ | ✓ |  |  |
| Adverse events |  |  | ✓ | ✓ | ✓ | ✓ | ✓ |  | ✓ | ✓ | ✓ | ✓ | ✓ |  |
| Body weight, BP, pulse | ✓ | ✓ | ✓ | ✓ | ✓ | ✓ | ✓ | ✓ | ✓ | ✓ | ✓ | ✓ | ✓ | ✓ |
| Serum biochemistry | ✓ | ✓ | ✓ | ✓ | ✓ | ✓ | ✓ | ✓ | ✓ | ✓ | ✓ | ✓ | ✓ | ✓ |
| Spot urine for proteinuria | ✓ | ✓ | ✓ | ✓ | ✓ | ✓ | ✓ | ✓ | ✓ | ✓ | ✓ | ✓ | ✓ | ✓ |
| Urinary gene expression |  | ✓ |  | ✓ |  |  | ✓ | ✓ |  | ✓ |  |  | ✓ |  |
| Serum inflammatory marker |  | ✓ |  | ✓ |  |  | ✓ | ✓ |  | ✓ |  |  | ✓ |  |
| Fasting glucose, lipid |  | ✓ |  |  |  |  | ✓ |  |  |  |  |  | ✓ |  |

*if LMP > 28 days
